# Purification of Hollow Sporopollenin Microcapsules from Sunflower and Chamomile Pollen Grains

**DOI:** 10.3390/polym13132094

**Published:** 2021-06-25

**Authors:** Jose Manuel Ageitos, Sandra Robla, Lorena Valverde-Fraga, Marcos Garcia-Fuentes, Noemi Csaba

**Affiliations:** Centre for Research in Molecular Medicine and Chronic Diseases (CiMUS), Department Pharmacology, Pharmacy and Pharmaceutical Technology, School of Pharmacy, Universidade de Santiago de Compostela, 15782 Santiago de Compostela, Spain; josemanuel.ageitos@gmail.com (J.M.A.); sandra.robla@outlook.es (S.R.); lorena.valverde@rai.usc.es (L.V.-F.); marcos.garcia@usc.es (M.G.-F.)

**Keywords:** sporopollenin, microcapsules, pollen, sunflower, chamomile, FTIR, SEM

## Abstract

Pollen grains are natural microcapsules comprised of the biopolymer sporopollenin. The uniformity and special tridimensional architecture of these sporopollenin structures confer them attractive properties such as high resistance and improved bioadhesion. However, natural pollen can be a source of allergens, hindering its biomedical applicability. Several methods have been developed to remove internal components and allergenic compounds, usually involving long and laborious processes, which often cannot be extended to other pollen types. In this work, we propose an abridged protocol to produce stable and pristine hollow pollen microcapsules, together with a complete physicochemical and morphological characterization of the intermediate and final products. The optimized procedure has been validated for different pollen samples, also producing sporopollenin microcapsules from *Matricaria* species for the first time. Pollen microcapsules obtained through this protocol presented low protein content (4.4%), preserved ornamented morphology with a nanoporous surface, and low product density (0.14 g/cm^3^). These features make them interesting candidates from a pharmaceutical perspective due to the versatility of this biomaterial as a drug delivery platform.

## 1. Introduction

Pollen grains are protective microcapsules of the male gametes of seed plants, involved in dispersion and adhesion, as well as recognition, during pollination and fertilization processes [1]. These natural microcapsules are composed by several layers of different lipidic and polymeric materials that confer them unique, plant-specific morphologies [2]. The terminology of pollen wall layers is heterogeneous in the literature and is usually based on the different staining affinity of the biomaterials [3]. Basically, three main layers can be distinguished: first, the external coating, named pollenkitt; followed by the multi-layered pollen-wall (exine and intine); and finally, the inner sporoplasm, where the cell gamete is located [4]. The exine is mainly composed by sporopollenin, one of the most resistant biopolymers found in nature, still part of 500-million-year-old sedimentary rocks [5,6]. This biomaterial is considered a keystone in the adaptation of early aquatic plant gametes to terrestrial life [7]. Sporopollenin is composed of carbon, oxygen, and hydrogen and is constituted of long, highly cross-linked biopolymer chains [7]. In addition to its unique chemical composition, pollen exine has a complex tridimensional structure with ornaments and nanopores, naturally designed for interacting with different biological surfaces, such as insect cuticula, animals, and plant tissues.

Synthetic microcapsules often present variability in size and morphology during their production. In contrast, pollen grains can be considered natural, monodisperse nanostructured microcapsules with highly resistant and specific three-dimensional morphologies that, so far, are difficult to replicate with current methods [8]. Pollen structure and composition are dependent on the dispersal pathway of the species (wind, water, insects, animals) in order to protect the male gametophyte best from external environmental conditions. These unique properties have already drawn researchers’ attention [2]; notwithstanding, it is well known that most pollens contain allergens that constitute a potential risk to human health [1,9]. A few protocols have already been established for the elimination of such components and/or the chemical extraction of sporopollenin from fern spores and pollen grains [10,11,12] to encapsulate drugs [13,14], lipids [15], cells [16], proteins [17,18], or chemical catalysts [19,20]. Such procedures usually involve laborious processes using harsh reagents (e.g., strong acids and/or bases) and prolonged incubation times that can alter the ultrastructure of the pollen grain [10,12].

In addition, the above-mentioned pollen species are generally characterized by simple morphology and produce thin microcapsules with limited applicability. Looking for alternatives, sunflower (*Helianthus annuus*) pollen has been recently drawing attention due to its widespread cultivation, flower size, and high pollen production with low allergenicity [10,11,12]. Chamomile (*Matricaria chamomilla*, sym. *M. recutita*) belongs to the same family as sunflower and is one of the most important medicinal plants [21,22]. Chamomile pollen is smaller than that of sunflower (13–25 µm vs. 26–50 µm) [23,24] and has a highly porous surface. Despite its interesting size and structure, which make it an ideal candidate for producing sporopollenin microcapsules, this pollen has not yet been explored for this purpose.

**Figure 1:**
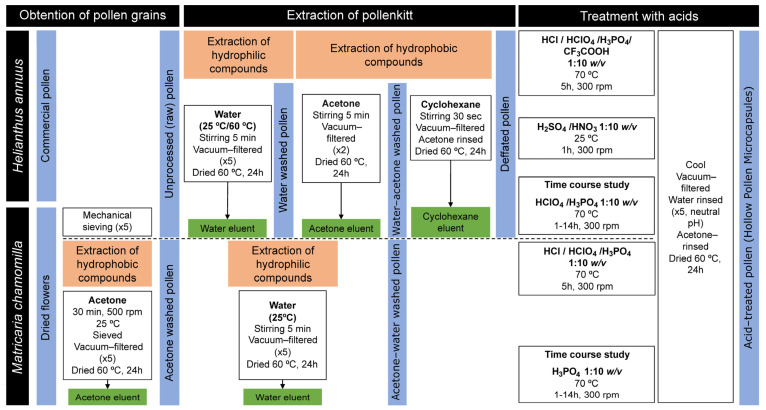
Processing scheme for the different pollen samples.

Taken together, the aim of our study has been the establishment of a simplified protocol for the obtention of hollow pollen microcapsules, employing sunflower pollen as reference (Figure 1), and the evaluation of its the suitability for chamomile pollen purification. Each step of the process has been studied in detail by several independent techniques to characterize the pollen, as well as the extracted products. To the best of the authors’ knowledge, this is the first comparative morphological and physicochemical characterization of sporopollenin microcapsules throughout their sequential chemical purification steps, as well as the first report on the production of hollow sporopollenin microcapsules from chamomile pollen.

## 2. Materials and Methods

### 2.1. Materials

Sunflower pollen (*H. annuus*) was obtained from Control Bio (El Alquián, Spain) and Pharmallerga (Lisov, Czech Republic). Chamomile (*M. chamomilla*) dried plant and flower were purchased from Soria Natural (Soria, Spain). Cyclohexane, orthophosphoric acid (H_3_PO_4,_ 85%), perchloric acid (HClO_4,_ 70%), hydrochloric acid (HCl, 37%), and sulfuric acid (HCl, 98%) were purchased from Merck KGaA (Darmstadt, Germany). Trifluoroacetic acid (CF_3_COOH, 99%), nitric acid (HNO_3_, 65%), and further standard chemicals were obtained from Sigma-Aldrich (Madrid, Spain). Organic solvents were HPLC grade, and all other products used were of reagent grade purity or higher. All chemicals were used as received without further purification.

### 2.2. Treatment of Sunflower Pollen

Pollen of *H. annuus* was treated in several consecutive washing steps to remove the external pollenkitt coating of the sporopollenin (Figure 1), as well as internal cellular components.

#### 2.2.1. Extraction of the Pollenkitt of Sunflower Pollen

Sunflower pollen was treated with water at different temperatures (25 or 60 °C) to extract hydrophilic compounds from pollenkitt. For this purpose, 2 g of pollen were mixed with 40 mL of water and vortexed for 5 min. Afterwards, the suspension was vacuum filtered. First, filtration eluant was recovered, frozen, and lyophilized. Pollen was subsequently resuspended in 40 mL of water and filtered in the same conditions. Water extraction process was repeated in a total of five washes. Water-washed pollen was recovered by filtration and dried at 60 °C for 24 h or until no weight variation was observed.

Water-treated pollen was resuspended in 20 mL of acetone, homogenized for 5 min by vortex, and vacuum filtered (two times). Acetone-treated pollen was recovered by filtration and dried again at 60 °C for 24 h. For the obtention of defatted sunflower pollen, acetone-treated pollen was resuspended in 20 mL of cyclohexane [25] and vortexed for 30 s. Cyclohexane-treated pollen was recovered by filtration, rinsed with acetone, and left overnight in a fume hood. Afterwards, it was dried at 60 °C for 24 h and stored in a desiccant chamber. Acetone and cyclohexane eluents were concentrated by rotary evaporation for further analysis.

#### 2.2.2. Elimination of Sporoplasm, Production of Hollow Sunflower Pollen with Acids

For the obtention of hollow sunflower pollen [10], several acids were assayed as follows. On one hand, sunflower pollen was incubated with concentrated acid (HCl, HClO_4_, H_3_PO_4_, CF_3_COOH) at 1:10 ratio (*w*/*v*) in a bath at 70 °C with gentle stirring at 300 rpm. For the time course study, defatted sunflower pollen was incubated with HClO_4_ and H_3_PO_4_ from 1 to 14 h under the same conditions as above. The same pollen to acid ratio (1:10 *w*/*v*) was employed with H_2_SO_4_ or HNO_3_ at 25 °C for 1h under gentle stirring (300 rpm). Afterwards, solutions were cooled, and acid-treated pollen was recovered by filtration in each case. The filtrate was rinsed with water until the pH of the pollen grains became neutral (five washes with 50 mL of water) and rinsed with acetone. Finally, acid-treated (hollow) pollen was dried at 60 °C for 24 h and stored in a desiccant chamber until use.

### 2.3. Treatment of Chamomile Dried Plant and Flower

A protocol for the purification of chamomile pollen was designed using dried flowers as starting material, since no available commercial source was found for this specific pollen.

#### 2.3.1. Purification of Pollen from Dried Chamomile

Several strategies were assayed for the extraction of pollen from dried chamomile with the purpose of achieving optimal conditions. For physical extraction of unprocessed pollen, 1 g of dried chamomile was sieved through 50 µm and 30 µm mesh fitted stainless steel sieves of 8 cm diameter. Five cycles of sieving were performed, recovering the unprocessed pollen from the lower reservoir. Process was repeated for a total of 15 g of dried plant and flowers.

As another strategy, the pollen was extracted from 15 g of dried chamomile flowers by infusion with 500 mL of acetone during 30 min at 500 rpm and 25 °C. Acetonic infusion was filtered through 50 µm and 30 µm mesh fitted stainless steel sieves followed by a paper filter, employing vacuum. Acetone eluant was recovered and added to the acetone-treated chamomile. Incubation and filtering process were repeated until no visible pollen was recovered from the acetonic infusion (i.e., five extractions). Pollen was then rinsed with fresh acetone, dried at 60 °C for 24 h and stored in a desiccant chamber until use. Acetone eluant was concentrated by rotary evaporation and the concentrated extract was recovered for analysis.

#### 2.3.2. Extraction of Pollenkitt of Chamomile Pollen

Unprocessed chamomile pollen obtained by mechanical sieving, was treated as described for sunflower pollen (Section 2.2), maintaining the 1:10 ratio (*w*/*v*). In the case of pollen already extracted with acetone, only the extraction process of hydrophilic compounds was performed, as described above.

#### 2.3.3. Elimination of Sporoplasm, Production of Hollow Chamomile Pollen with Acids

Acetone–water-treated pollen was incubated with concentrated acid (HCl, HClO_4_, H_3_PO_4_) at 1:10 ratio (*w*/*v*) in a bath at 70 °C for 5 h under gentle stirring at 300 rpm. For the time course study, acetone–water-treated pollen was incubated with H_3_PO_4_ at 1:10 ratio (*w*/*v*) for 1 to 14 h under the same conditions. Filtering and washing processes were performed as described in Section 2.2.2.

### 2.4. Analytical Measurements

All analytical measurements were performed at the analytical facilities of the University of Santiago de Compostela (RIAIDT).

#### 2.4.1. Fourier Transform Infrared Spectroscopy (FTIR)

FITR technique was employed to explore the different functional groups present in the pollen samples [26] after each purification step as well as in the obtained eluates. FTIR spectra were recorded in a VARIAN FT-IR 670 (Varian Inc. Scientific Instr., Palo Alto, CA, USA) equipped with an attenuated total reflectance (ATR) accessory (GladiATR, PIKE technologies, Madison, WI, USA) from 400 to 4000 cm^−1^ at 4 cm^−1^ resolution using 64 scans.

#### 2.4.2. Elemental CHN Analysis

The CHN analyses were carried out using an EA1108 CHNS-O Elemental Analyzer (Fisons Instrument, Mt Pleasant, NJ, USA). Protein content of samples was estimated using the percent of nitrogen content with a conversion factor of 6.25 [10].

#### 2.4.3. Scanning Electron Microscopy (SEM) Analysis

Pollen grains were analyzed in a field emission scanning electron microscopy (Fesem Ultra Plus, Zeiss, Jena, Germany). Samples were deposited in a carbon tape and covered with iridium. Micrographs were recorded with an acceleration voltage of 3.00 kV at different magnifications (5000×, 20,000× or 30,000×).

#### 2.4.4. Confocal Laser Scanning Microscopy (CLSM)

Internal and external structures of treated pollen grains were studied by CLSM (SP5 Leica AOBS-SP5, Leica Biosystems Nussloch GmbH, Wetzlar, Germany) employing the natural autofluorescence of pollen (λ_ex_: 405 nm, λ_em_: 414–479 nm; λ_ex_: 492 nm, λ_em_: 505–554; λ_ex_: 561 nm, λ_em_: 571–635 nm) [27].

#### 2.4.5. Determination of Pollen Density and Number of Grains per Unit of Mass

Tapped density of pollen samples was determined by gravimetry of five different volumes of pollen with a graduated cylinder. Pollen was manually tapped for 5 min until no variations in the volume were observed. Tapped density was measured with three different batches per group. Number of grains per unit of mass was calculated using a haemocytometer (Neubauer chamber, Paul Marienfeld GmbH & Co. KG, Lauda-Königshofen, Germany) [28], where 1 mg of pollen sample was resuspended in 1 mL of water and immediately counted by optical microscopy. Number of grains per unit of mass was measured in triplicate employing three batches for each sample.

#### 2.4.6. Thermal Gravimetric Analysis (TGA)

The thermal properties of the different pollen samples were investigated using TGA analyses (TGA/DSC 1–Thermogravimetric Analyzer; Mettler Toledo GmbH, Schwarzenbach, Switzerland) performed in N_2_ atmosphere. Samples were analyzed in an alumina pan at 10 °C/min as heating rate. Differential thermal gravimetry analysis was calculated employing the OriginPro 2017 software (Origin Lab Corporation, Northampton, MA, USA).

### 2.5. Statistical Analysis

Statistical significance of the difference between the means was determined by analysis of variance (ANOVA) followed by pairwise multiple comparison using Tukey’s HSD method. Pair comparisons were evaluated by Student t-tests. All tests were performed with Statgraphics centurion XVIII software (Statgraphics Technologies, Inc., The Plains, VA, USA). Differences were considered significant at *p* < 0.05.

## 3. Results and Discussion

### 3.1. Extraction of the Pollenkitt: Sunflower Pollen

**Figure 2:**
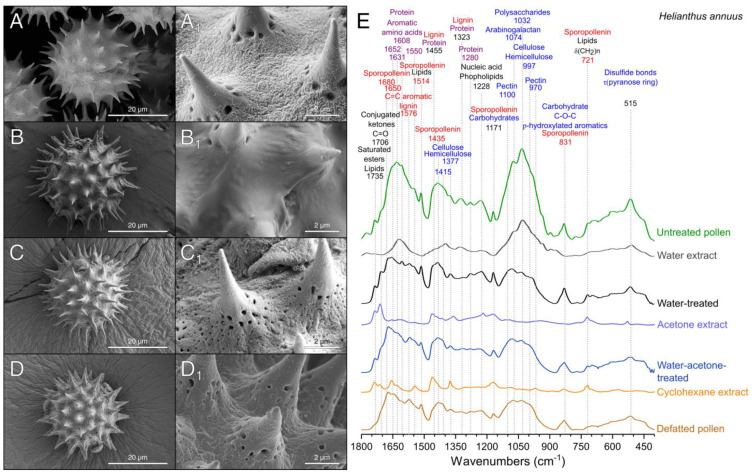
Scanning electron micrographs of sunflower pollen samples (**A**–**D**) (**A**) Untreated pollen. (**B**) Water-treated pollen. (**C**) Water-treated pollen after treatment with acetone. (**D**) Defatted sunflower pollen. (**E**) ATR-FTIR spectra of sunflower pollen samples and extracts obtained during the purification depicting characteristic bands.

Sunflower pollen (Figure 2) was selected for the optimization and validation of the purification method because of its echinate and tricolporate structure, which makes it an interesting candidate from a technological standpoint. Sunflower pollen grains are composed by a multilayer structure covered by an external layer named pollenkitt. This layer fills the nanopores on the surface (Figure 2A) and is prone to contain environmental contaminants and allergens, such as coat proteins [25]. To remove the pollenkitt, a sequential treatment was designed based on the different solubility of coating materials and sporopollenin [29]. To study the components removed on each step, both the purification extracts and pollen were studied by SEM and FTIR analyses (Figure 2E), techniques widely employed for the identification of pollen species and biomaterials [30,31,32].

**Figure 3:**
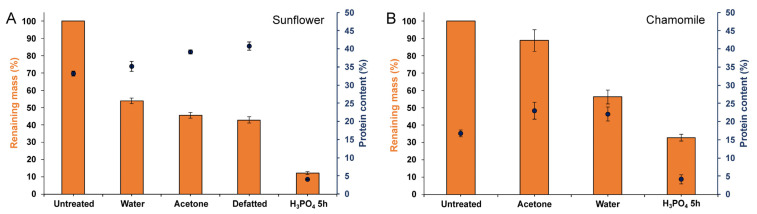
Remaining mass (bars) and protein content (% of remaining mass, dots) of pollen samples during purification process. (**A**) Sunflower. (**B**) Chamomile. Error bars represent the standard deviation of the mean (n = 3).

Untreated samples presented the typical morphology of raw sunflower pollen with the surface completely coated with pollenkitt (Figure 2A), as well as their characteristic FTIR spectrum [26] (Figure 2E). We analyzed sunflower pollen from two different origins: Spain, and Czech Republic. Both samples presented the same FTIR bands but with variations in their relative intensities, particularly for the signals assignable to proteins and polysaccharides (Figure S1A). Water-soluble and debris materials from the exterior of raw pollen (Figure 2A) were more efficiently extracted with purified water at 25 °C than at 60 °C, possibly due to the denaturalization of proteins at temperatures over 55 °C. After this step, the spectra of sunflower pollen from both origins were similar (Figure S1A). The water-washing treatment mainly extracted carbohydrates [33,34] and proteins [35,36] (Figure 2E), which represented 46% of the raw pollen mass (Figure 3A).

As expected, this treatment did not fully eliminate coating materials, and therefore, nanopores of the pollen wall were still not clearly uncovered (Figure 2B). This preliminary water-washing step is unfrequently used in the literature, where it is more common to treat pollen directly with organic solvents. However, polysaccharides and proteins are poorly soluble in organic solvents [37], which leads to suboptimal purification of the samples. Once water-soluble subproducts were removed, the extraction of the lipophilic compounds of pollenkitt was assayed using a combined sequential treatment of acetone and cyclohexane. Prolonged incubation in hot acetone is another widely employed method to produce defatted pollen [27,38,39], but similar results could be obtained with shorter extractions processes [40]. In this study, the short washing with acetone at 25 °C (Figure 2C) extracted α-linolenic acid (3008, 1220, 721, 606, 486 cm^−1^), lauric acid (2922 and 2852 cm^−1^), stearic acid (1113 cm^−1^), and other lipids (1711, 1462, 1415 cm^−1^) [41,42]. The bands of compounds such as aliphatic esters (1173 cm^−1^) [43], carboxylic groups (970 and 948 cm^−1^) [34,44], and phenols (1620 cm^−1^) [45] were also present in the spectra of the acetone extract. As compared to hot acetone extraction, our low temperature process could be advantageous as it should reduce any thermal alteration of the product. After this step, sunflower pollen became white, indicative of the removal of flavonoids and carotenoid pigments [26], and product mass was reduced by approximately 8% (Figure 3A). Acetone washing was unable to eliminate saturated lipids and proteins, as shown by FTIR bands at 1735 cm^−1^ (Figure 2E). To improve the extraction of these insoluble compounds [25], an additional cyclohexane washing step was performed. This allowed the elimination of surface components without causing morphological changes in the exine (Figure 2D). Lipid and protein extraction (Figure 2E) [25] was confirmed by the disappearance of the bands at 1735 cm^−1^ (saturated esters [45]), 1655, 1627, 1608, 1547, and 1455 cm^−1^ (proteins [35,36]), and 1455, 720 cm^−1^ (lipids [41]). The analysis of the cyclohexane extract (Figure 2E) showed the presence of additional compounds, such as pectin, with a signal at 970 cm^−1^ [46]. Cyclohexane extraction represented a further mass reduction of 2.6% from the raw pollen mass (Figure 3A). The accumulated percent of mass corresponding to the lipid phase of the pollenkitt (~10%, Figure 3A) was in line with the lipid content previously reported for sunflower pollen [26,47]. After treatment with cyclohexane, the bands assigned to sporopollenin [48] became evident in the spectra (Figure 2E), indicating the successful cleaning of the pollen surface.

Albeit not abundant in *H. annuus*, proteins and glycoproteins are the principal allergenic compounds found in pollen [49]. Therefore, protein elimination is a critical point for pollen purification when considering pharmaceutical applications. The protein content of the samples was calculated from the nitrogen content determined by CHN elemental analysis (Figure 3A). Protein percent of raw sunflower pollen was initially ~33 wt%, and this fraction rose to ~40 wt% after pollenkitt elimination. This value was higher than that previously reported for commercial defatted sunflower pollen grains (~32 wt% of proteins) [10,17]. The relative increase in the protein content of defatted sunflower pollen, as compared with the untreated one, can be explained by the elimination of lipids and polysaccharides, rich in C and H but without N, and by the reduction of the relative mass by dehydration. Protein removal from this product, leading to hollow sporopollenin microcapsules, was undertaken in a subsequent step (Section 3.3).

### 3.2. Extraction of Pollenkitt: Chamomile Pollen

Considering the results obtained after the treatment of sunflower pollen, the applicability of the method was also tested for *M. chamomilla*, a widely studied medicinal plant, with an echinate pollen grain of smaller size (13–25 µm vs. 26–50 µm [23,24]). In contrast to sunflower, it is unusual to find commercially available *M. chamomilla* pollen. However, their dried flowers are universally available, and thus, we devised a cleaning protocol starting from this source (Figure 1).

**Figure 4:**
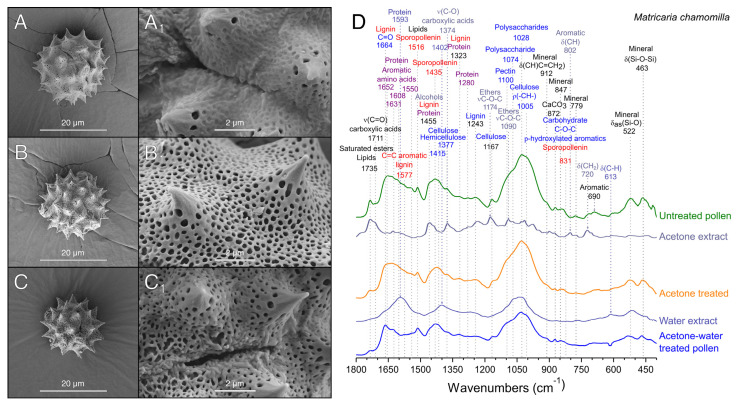
Scanning electron micrographs of chamomile pollen samples (**A**–**D**) (**A**) Untreated pollen. (**B**) Pollen treated with acetone. (**C**) Acetone–water-treated pollen. (**D**) ATR–FTIR spectra of pollen samples and extracts obtained during the elimination of pollenkitt.

The isolation of pollen by mechanical sieving from dried flowers was only feasible for small batches, yielding a 0.2 ± 0.1% of pollen. This indicated an extraction below 10% since it is estimated that pollen represents the 2.5% of the weight of dried *Matricaria* sp. flowers [50]. Although still suboptimal, this extraction technique was the only one that allowed the obtention of pollen with intact pollenkitt coating (Figure 4A). Due to the low productivity of mechanical sieving, several alternative methods for pollen recovery were assayed, such as initial pollen extraction with water or acetone. Water extraction of chamomile pollen from dried flowers produced the release of mucilage [21] that obturated the paper filters and hindered recovery, being eventually discarded as a pollen extraction method. On the other hand, when acetone was employed (Figure 1), pollen was easily recovered by filtration, allowing for scaling-up the process and achieving a pollen mass yield of 2.3 ± 0.1% with respect to the dried flowers (88.9 ± 6.2% *w*/*w* recovery, Figure 3B), close to the above-mentioned theoretical value [50]. This difference in the extraction efficacy can be attributed to the poor solubility of mucilage in acetone.

**Figure 5:**
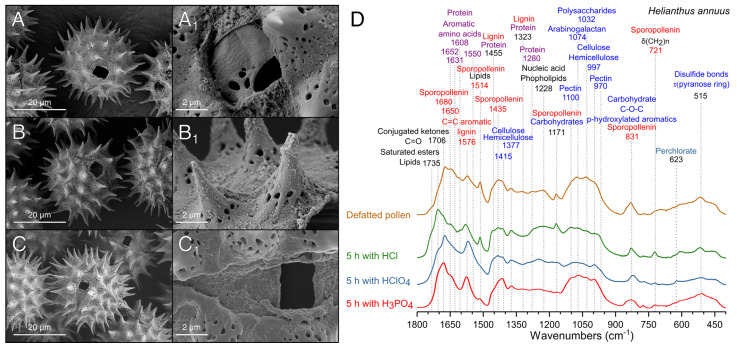
Scanning electron micrographs of defatted sunflower pollen treated with different acids. (**A**) HCl. (**B**) HClO_4_. (**C**) H_3_PO_4_. (**D**) ATR-FTIR spectra of pollen samples after 5 h treatment.

As compared to sunflower pollen grains (Figure 2A), chamomile pollen is smaller, and its exine layer is less covered with pollenkitt (Figure 4A_1_). Due to these inherent differences, the protein content of raw chamomile pollen was found to be significantly lower (16.6 ± 0.9 wt%, Figure 3B) than that of raw sunflower pollen (Figure 3A). The FTIR spectra of unprocessed chamomile pollen (Figure 4D) was similar to sunflower (Figure 2E) except for changes in relative band intensities attributed to lipids, polysaccharides, cellulose, and the presence of minerals in chamomile. This similarity could be expected as both plants belong to the Asteraceae family [51]. The main differences were the absence of a 1774 cm^−1^ band of esterified pectin [52] present in sunflower (Figure 2E) and the presence of several bands corresponding to minerals in chamomile pollen (Figure 5B) [53], which were probably debris derived from flower harvesting.

After filtration and recovery of the pollen, the yellow eluant was concentrated in a rotavapor, and the recovered acetone was recycled for further pollen extraction. The extract was analyzed by FTIR (Figure 4D), and the presence of bands previously described for chamomile oil, such as ethers (1174, 1090 cm^−1^) and carboxylic acids (1374 cm^−1^), was found [54,55]. Therefore, we expect that this subproduct could be a starting point for the purification of therapeutically significant bioactive compounds [21] in the future.

Untreated chamomile pollen had its exine pores filled with pollenkitt (Figure 4A), but these became clearly visible after acetone extraction (Figure 4B). The protein content of chamomile pollen treated with acetone was 22.9 ± 2.3 wt%, significantly higher than that of raw pollen (Figure 3B). This can be attributed to the elimination of hydrocarbon-rich compounds, as previously discussed for sunflower pollen in Section 3.1. Acetone extracted pollen was washed with water as described above, resulting in a slight reduction in average size (16 ± 3%) (Figure 4C). The water extract exhibited FTIR bands assignable to polysaccharides and proteins, and accordingly, the pollen spectrum showed a decrease in their corresponding bands (Figure 4E). The protein content remained similar (22.0 ± 1.9 wt%, no significant difference), although pollen mass was reduced by ~33% (Figure 3B). FTIR analysis showed that the main components extracted with water treatment consisted of proteins and polysaccharides (Figure 4E). The spectra of the defatted chamomile pollen (Figure S1B), treated with the protocol developed for sunflower (Figure 1), showed a less efficient removal of polysaccharides than the pollen only treated with acetone and water, suggesting that the use of cyclohexane is not required in this case. Hence, the following experiments were performed with acetone-water-treated pollen.

### 3.3. Comparison of Different Acid Treatments on the Purification of Pollen

Sporopollenin is generally described as a biopolymer highly resistant to acidolysis [6]; however, harsh acidic treatment can affect its ultrastructure [10]. Therefore, acidolysis processes were optimized herein to maximize solubilization and removal of internal components (proteins, polysaccharides, and lipids) while preserving the sporopollenin and pollen structure. Sunflower pollen presented different sensibility depending on the acid treatment used. For instance, CF_3_COOH was unable to penetrate the pollen wall after 5 h at 70 °C while H_2_SO_4_ and HNO_3_ completely destroyed their structure, even after 1 h incubation at room temperature. Among the assayed acids, HCl, HClO_4_, and H_3_PO_4_ were the most effective removing the cellular content without affecting sporopollenin morphology and chemical composition (Figure 5). Treatment with HCl was unable to produce homogeneous hollow pollen grains (Figure 5A) but decreased protein and polysaccharide content (Figure 5D). The intense band at 1705 cm^−1^ suggests the formation of hydrochloride salts [56] and an increase in the formation of conjugated ketones (C=O stretching) after the degradation of lignin [57], which might indicate that HCl produced chemical modifications to the pollen structure and the degradation of the intine.

HClO_4_ is a strong acid with oxidizing properties at high temperatures that has not been previously applied for sporopollenin purification. However, it has been used in the extraction and digestion of polysaccharides [39], polyamines [58], and proteins [59] in bee pollen and plant tissues. To study the suitability of this acid for pollen purification, a time course study was carried out with sunflower pollen using FTIR analysis and SEM images to evaluate the process (Figure S2). Results indicated that 5 h of treatment (Figure 5B) produced a cleaner intine as compared with shorter treatments (Figure S2). The percent of protein of HClO_4_-treated samples decreased up to 9 wt% at 5 h, a similar value to that reported for samples treated for 20 h in HCl (~10 wt%) [10]. SEM images evidenced that HClO_4_ treatment left residues on the exine surface (Figure 5B_1_), probably perchlorates that could also be detected through their FTIR band at 623 cm^−1^ [60] (Figure 5D and Figure S3B). Although HClO_4_ can be considered an alternative for the acid treatment of pollen, it produces chemical modifications in the pollen.

H_3_PO_4_ yielded excellent results in the production of sunflower hollow pollen microcapsules (Figure 5C), as the relative intensity of the FTIR bands assignable to proteins (1631, 1608, 1550, 1455 cm^−1^) and carbohydrates (1435, 1323, 1030 cm^−1^) decreased sharply (Figure 5D) [41]. Treatment with H_3_PO_4_ did not reduce bands of hemicellulose and cellulose (Figure 5D), indicative of the preservation of intine, while the protein content after this treatment decreased to 4.4 ± 0.8 wt%. After H_3_PO_4_ treatment, the hollow sunflower microcapsules of different origin were indistinguishable in their FTIR spectra (Figure S1). Treatment with H_3_PO_4_ allowed the efficient production of hollow pollen, stable to dehydration or heating processes, without the requirement of additional neutralization steps. Optimization of this procedure is discussed in detail in Section 3.4.

**Figure 6:**
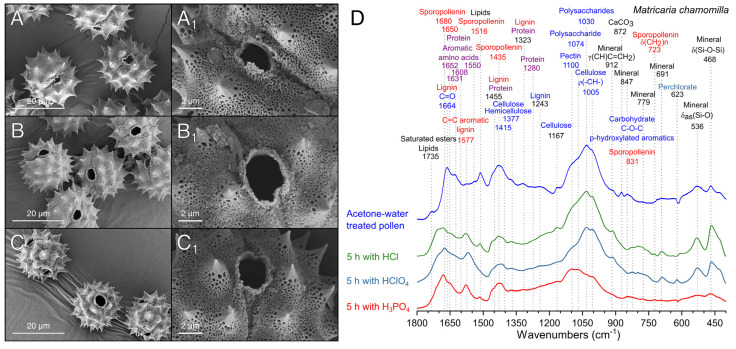
Scanning electron micrographs of acetone–water-treated chamomile pollen incubated with different acids. (**A**) HCl. (**B**) HClO_4_. (**C**) H_3_PO_4_. (**D**) FTIR spectra of pollen samples after 5 h treatment.

In view of the results obtained with sunflower pollen, chamomile pollen was only treated with the best performing acids, i.e., HCl, HClO_4_, and H_3_PO_4_ (Figure 6). The treatment with HCl for 5 h was not able to produce a homogeneous aperture of pollen (Figure 6A); however, protein bands reduced their relative intensity (Figure 6D), while bands related to polysaccharides and impurities remained unaltered.

HCl-treatment produced a shoulder at 1706 cm^−1^ in chamomile pollen, which, as observed in sunflower pollen (Figure 5D), is indicative of the formation of carboxylic acids [61]. The incubation with HClO_4_ produced the aperture of the pollen (Figure 6B), but the signals of polysaccharides and mineral impurities did not decrease (Figure 6D). Perchlorate deposits were evident on the surface of the exine (Figure 6B_1_) and in the FTIR spectra (Figure 6D). The treatment with H_3_PO_4_ produced a uniform aperture of chamomile pollen grains (Figure 6C) and a drastic reduction of polysaccharides and protein bands (Figure 6D). The bands of mineral compounds present in chamomile pollen also disappeared from the FTIR spectra after H_3_PO_4_ treatment (Figure 6D). The efficacy of H_3_PO_4_ in the removal of minerals can be explained by its high reactivity forming soluble salts with metals such as calcium, aluminum, or iron [62], elements commonly found in minerals [53]. The treatment employed on sunflower showed similar results in chamomile, confirming that sunflower pollen was a good initial model that allowed the extension of the study to other members of the Asteraceae family.

### 3.4. Optimization of the Treatment with H_3_PO_4_

**Figure 7:**
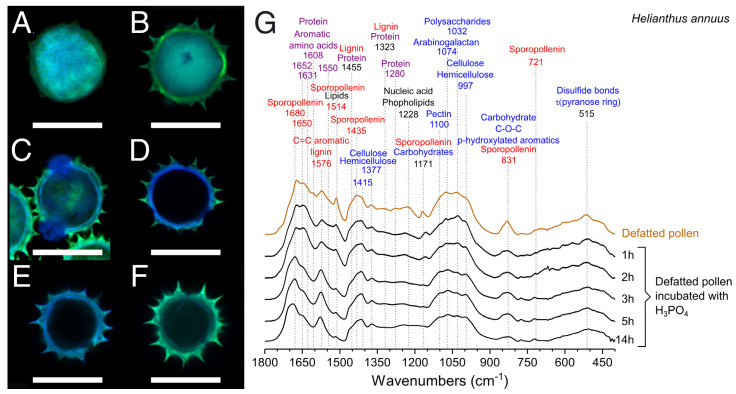
Confocal laser scanning micrographs of sunflower pollen samples. (**A**) Untreated pollen. (**B**) Defatted pollen. (**C**–**F**) Defatted pollen treated with H_3_PO_4_, 70 °C. (**C**) 2 h. (**D**) 3 h. (**E**) 5 h. (**F**) 14 h. Green channel: Exine. Blue channel: Intine and sporoplasm. (Scale bars represent 30 µm). (**G**) FTIR spectra of defatted pollen treated with H_3_PO_4_ at 70 °C for different time points.

Since H_3_PO_4_ showed the best results in the production of hollow pollen microcapsules for both sunflower and chamomile (Figure 5C and Figure 6C), we analyzed the time course of the purification process by confocal laser scanning microscopy (CLSM) and FTIR. CLSM has been employed as a reference technique in the characterization of sporopollenin microcapsules [17,18,27,45] due to the strong natural autofluorescence of pollen grains, derived from sporopollenin, flavonoids, and carotenoids [63]. Untreated sunflower pollen (Figure 7A) showed a strong overlapping fluorescence, hindering a clear distinction of the pollen layers. Defatted sunflower pollen presented a more defined autofluorescence pattern, with an internal cavity with a strong fluorescence (Figure 7B), due to the proteins and coenzymes present in the sporoplasm [63].

One hour of treatment with H_3_PO_4_ did not lead to large differences on defatted sunflower pollen (data not shown), but the permeabilization of the outer layer was observed after 2 h of treatment (Figure 7C), when sporoplasm components accumulated into the apertures. Definitive disappearance of the sporoplasm was not evident until 3 h (Figure 7D). At this point, fluorescent compounds remaining in exine and intine allowed a clear distinction between those layers. Pollen incubated with H_3_PO_4_ for 5 h (Figure 7E) had lower overall fluorescence but similar levels of autofluorescence of exine and intine. The lack of internal fluorescence confirmed the elimination of the sporoplasm [10]. For samples incubated for 14 h in H_3_PO_4_ (Figure 7F), the intine fluorescence signal decreased, and it appeared clearly detached from exine. This process led to unstable and fragile structures [64]. Despite the morphological changes, during the first 2 h of treatment there were no differences in the FTIR spectra as compared to defatted pollen. After 3 and 5 h of treatment with H_3_PO_4_, the bands of protein (1631, 1608, 1550, 1455 cm^−1^) and carbohydrates (1435, 1323, 1030 cm^−1^) virtually disappeared from FTIR spectra. After 14 h, the sporopollenin band at 1680 cm^−1^ [48] shifted to a broad band at 1690 cm^−1^, suggesting the oxidation of this polymer [65] (Figure 7G). The protein content of H_3_PO_4_-treated sunflower pollen decreased with the incubation time, but it was not until 3 h that this decrease was significant (9.1 ± 0.4 wt%). There was not a statistically significant difference in the protein content between 5 (4.4 ± 0.8 wt%) and 14 h treatment (4.5 ± 0.1 wt%).

Our results confirmed that 5 h of treatment with H_3_PO_4_ were enough to produce hollow pollen microcapsules [17] resistant to dehydration or heating processes. After 5 h, the mass of the pollen was reduced by a total 30% compared to defatted samples and represented 12% of the raw pollen (Figure 3A). Because of the treatment (Table S1), sunflower hollow pollen (0.13 ± 0.01 g/cm^3^, ~2.5 × 10^5^ grains/mg,) had approximately three times less density than defatted (0.38 ± 0.06 g/cm^3^, ~9.0 × 10^4^ grains/mg). Protein content and morphology of hollow sunflower pollen obtained by this method were comparable to those reported for 5 h [17] and 10 h [10,17] treatments with H_3_PO_4_ after intensive washing procedures.

**Figure 8:**
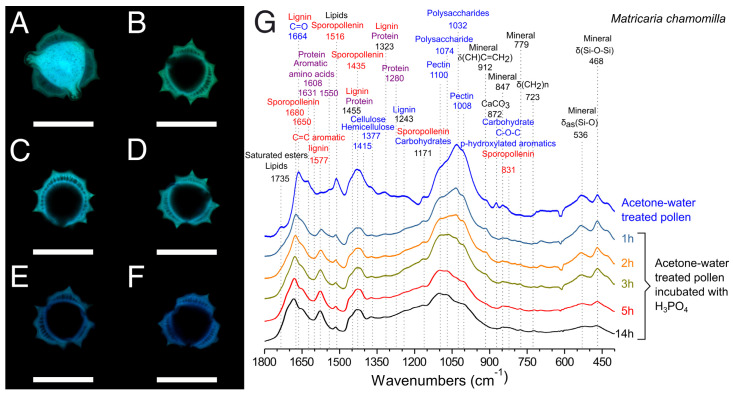
Confocal laser scanning micrographs of chamomile pollen samples. (**A**) Acetone–water-treated pollen. (**B**–**F**) Acetone–water-treated pollen incubated with H_3_PO_4_, 70 °C. (**B**) 1 h. (**C**) 2 h. (**D**) 3 h. (**E**) 5 h. (**F**) 14 h. Green channel: Exine. Blue channel: Intine and sporoplasm. (Scale bars represent 20 µm). (**G**) FTIR spectra of acetone–water-treated pollen treated with H_3_PO_4_, 70 °C for different time points.

**Figure 9:**
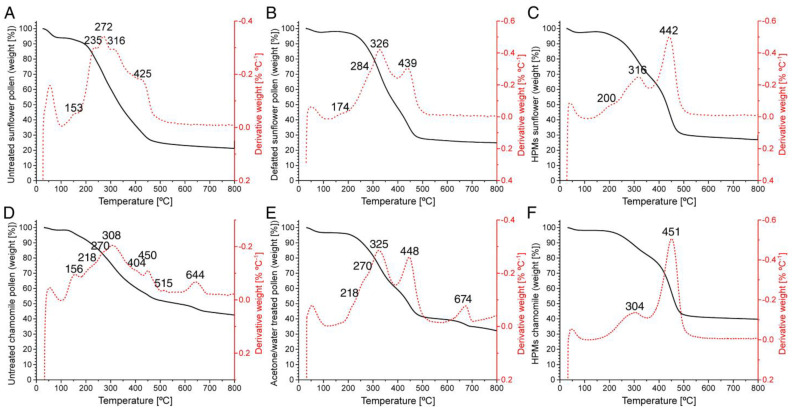
Thermogravimetric analysis of different pollen samples. (**A**) Untreated sunflower pollen. (**B**) Defatted sunflower pollen. (**C**) Sunflower hollow pollen microcapsules. (**D**) Untreated chamomile pollen. (**E**) Defatted chamomile pollen. (**F**) Chamomile hollow pollen microcapsules. Black line: Weight percentage (%). Red dotted line: Derived thermogravimetry.

Chamomile pollen exine has a columnar structure and it forms a thicker wall (Figure 8) than sunflower exine (Figure 7). Its diffuse autofluorescence did not allow for differentiation of the intine. In addition, chamomile pollen (Figure 8) was more sensitive to acid treatment than sunflower. After 1h of incubation, these pollen grains had already lost the sporoplasm fluorescence (Figure 8B), and the protein bands in FTIR (Figure 9G) reduced their relative intensity. The main difference observed as function of the incubation periods was polysaccharide content (1008, 1032 cm^−1^) [41], which decreased with time. FTIR spectra and CLSM images of 5 h and 14 h treatments were almost identical (Figure 8G); however, a shoulder in the FTIR at 14 h at 1710 cm^−1^ indicated the oxidation of the sporopollenin [41,66]. The protein content and the remaining mass of chamomile pollen treated with H_3_PO_4_ were also similar among the incubation periods, stabilizing at 4.0 ± 1.3 wt% after 5 h. In contrast to sunflower pollen, the mass of hollow chamomile pollen represented 33% of the grain mass (Figure 3B). Similar to sunflower pollen (Table S1), 5 h H_3_PO_4_-treated chamomile pollen had significantly lower density (0.14 ± 0.01 g/cm^3^, 1.0 × 10^6^ grains/mg) than defatted pollen (0.44 ± 0.02 g/cm^3^, 5.1 × 10^5^ grains/mg).

H_3_PO_4_ has been previously explored by other authors for the purification of hollow pollen grains. Nevertheless, those protocols involve extended incubation periods of defatting and acid treatment, with complex washing and neutralization processes, through multiple steps with organic solvents, acids, and bases [10,17,18,27]. The most common method for producing hollow pollen requires extensive treatments to eliminate acid residues, involving multiple washing steps, e.g., water (5×), acetone (2×), 2M HCl, water (5×), acetone and ethanol (2×) [10,17,18]. The method proposed in this work (Figure 1) drastically reduces and shortens treatment and washing procedures (6 h of defatting at high temperature, >10 steps after acidolysis vs. 10 min at room temperature and five steps of washing) and allowed the obtention of hollow pollen microcapsules with low protein content and preserved morphology, starting from pollen of different species and geographical origin.

### 3.5. Thermogravimetric Characterization of Hollow Pollen Platforms

Raw, defatted, hollow sunflower and chamomile pollen were analyzed by TGA (Figure 9, Table S2) to identify their thermal decomposition pattern. All samples showed a slight weight loss at low temperature (100 °C) due to the evaporation of absorbed water and humidity. Untreated sunflower pollen showed the highest variation of weight (Δ_W_) due to dehydration (Figure 9A), while defatted (Figure 9B) and hollow pollen followed a similar dehydration process (Figure 9C). Untreated sunflower pollen presented a thermal decomposition range (*T*_onset_) with five main stages (100–170 °C, volatile compounds; 170–250 °C, pectin and lipids; 250–300 °C, polysaccharides, proteins, lipids; 300–370 °C, cellulose, hemicellulose, sporopollenin; 370–480 °C, lignin, sporopollenin) [67,68,69,70], whereas defatted pollen and hollow pollen thermal decomposition was limited to three stages. Defatted pollen (Figure 9B, Table S2) presented the highest Δ_W_ with a temperature of decomposition (*T*_d_) of ~320 °C (*T*_onset_: 225–400 °C, Δ_W_: 40%), in the case of hollow pollen (Figure 9C, Table S2) the main Δ_W_ occurred at *T*_d_ ~440 °C (*T*_onset_: 360–530 °C, Δ_W_: 38%). Similar *T*_d_ has been reported for sporopollenin [70].

In the case of chamomile pollen, untreated pollen presented a complex decomposition pattern (Figure 9D, Table S2), indicating a higher percent of volatile compounds. Similarly, chamomile pollen had a lower percentage of lipids than sunflower pollen. In addition to that, the presence of *T*_d_ at 515 °C and 644 °C indicated the presence of mineral components in the sample, aligning with the results obtained by FTIR (Figure 4D). After cleaning of the pollenkitt, the thermal degradation pattern of chamomile pollen was simplified. The Δ_W_ attributable to sporopollenin increased up to 21%, but mineral debris was still present, as indicated by the *T*_d_ at 674 (Figure 9E).

Despite sunflower and chamomile pollen (Figure 5 and Figure 6) having differences in size (Table S1) and morphology (Figure 9A,D), the thermal degradation pattern of hollow chamomile pollen (Figure 9F, Table S2) was similar to sunflower (Figure 9C), indicating that, at this stage, both capsules have a very similar composition. In addition, after the purification with acid, the Δ_W_ attributable to mineral compounds was absent, indicating that this treatment successfully cleaned these impurities. These results suggest that the main components of hollow pollen microcapsules were cellulose and sporopollenin, while defatted pollen had a higher content of polysaccharides and proteins, supporting the results obtained with other techniques.

### 3.6. Comparison between Hollow Sporopollenin Microcapsules from Sunflower and Chamomile

Sunflower and chamomile belong to the same family, Asteraceae [51], characterized by pollen grains with three apertures and an echinate surface [23,24]. However, they present considerable differences regarding their size (Table S1). In fact, the inner cavity of hollow sunflower pollen is approximately the same size of the entire hollow chamomile pollen (Table S1). Interestingly, the apertures of both pollens are similar, with 5.2 ± 0.6 µm × 3.4 ± 0.4 µm for sunflower and 4.0 ± 0.1 µm × 2.9 ± 0.6 µm ± 0.4 µm for chamomile, respectively. On the other hand, even though they do not present significant difference in their densities (Table S1), the number of chamomile grains per unit of mass is one order of magnitude higher than sunflower (Table S1). This effect can be explained by their similar composition and different size (Figure 5D, Figure 6D, and Table S1). The structure of the exine is also different between species (Figure 7E and Figure 8E). Chamomile has a thicker columnar structure connected with the exterior by nanopores. In the case of sunflower, those pores are mainly located on the base of the spines (Figure 5C_1_), while in chamomile their distribution is homogeneous along the surface (Figure 6C_1_). These differences may affect the capacity for loading and releasing substances. Both hollow pollens have similar composition (Figure 5D, Figure 6D, and Table S1) and can be processed by the same procedure, but their different structural characteristics should play an important role in the loading and release of active ingredients. Moreover, hollow chamomile pollen could serve as an alternative to other small pollens with higher allergenicity, such as ragweed [38], for a variety of biomedical applications.

## 4. Conclusions

We have proposed an abridged protocol to produce pristine hollow sporopollenin microcapsules, as well as their complete physicochemical characterization throughout the purification process. The developed protocol can be applied to pollens from different species and/or origins, and it provides a faster and simplified procedure compared to those previously reported in the literature. By using this method, we also provide the first report on the production and characterization of purified pollen microcapsules from chamomile. Chamomile microcapsules present a nanoporous surface and a columnar inner structure of great interest for drug loading and release. In summary, the structure and characteristic of hollow sporopollenin microcapsules generated by the described purification methods make them promising candidates as devices for pharmaceutical applications.

## 5. Patents

M.G.-F. and N.C. are coinventors of the patents P201730151, P201730152 extended as PCT/ES2018/070092 “Purified pollen particles, their processing and application for the delivery of nanosystems”.

## Data Availability

Data presented in this study are available on request from the corresponding author.

## Supplementary Materials

The following results are available online at https://www.mdpi.com/article/10.3390/polym13132094/s1, Figure S1: ATR-FTIR spectra of pollen samples. A. Comparison spectra of sunflower pollen from Spain and Czech Republic. B. Spectra of chamomile pollen treated with extended extraction protocol, Figure S2: Time course study of the treatment of defatted sunflower pollen with HClO4. A. SEM images at different magnifications. B. ATR-FTIR spectra of pollen samples indicating the band assignment, Table S1: Principal characteristics of sunflower and chamomile pollen samples, Table S2: TGA results for sunflower and chamomile pollen samples.
