# Supplementary material for: Purification of Hollow Sporopollenin Microcapsules from Sunflower and Chamomile Pollen Grains
Source: Polymers (Basel). 2021 Jun 25;13(13):2094. doi: 10.3390/polym13132094 (PMC8271440; doi:10.3390/polym13132094)
Supplement: Supplementary file 1 [file polymers-13-02094-s001.zip › polymers-1240221-SI.pdf]

polymers

MDPI

Supporting material

Purification of Hollow Sporopollenin Microcapsules from Sunflower and Chamomile Pollen Grains

Jose Manuel Ageitos, Sandra Robla, Lorena Valverde-Fraga, Marcos Garcia-Fuentes and Noemi Csaba*

Centre for Research in Molecular Medicine and Chronic Diseases (CiMUS), Dept. Pharmacology, Pharmacy and Pharmaceutical Technology, School of Pharmacy, Universidade de Santiago de Compostela, 15782 Santiago de Compostela, Spain; josemanuel.ageitos@gmail.com (J.M.A.); sandra.robla@outlook.es (S.R.); lorena.valverde@rai.usc.es (L.V.-F.); marcos.garcia@usc.es (M.G.-F.)
*Correspondence: noemi.csaba@usc.es (N.C.)

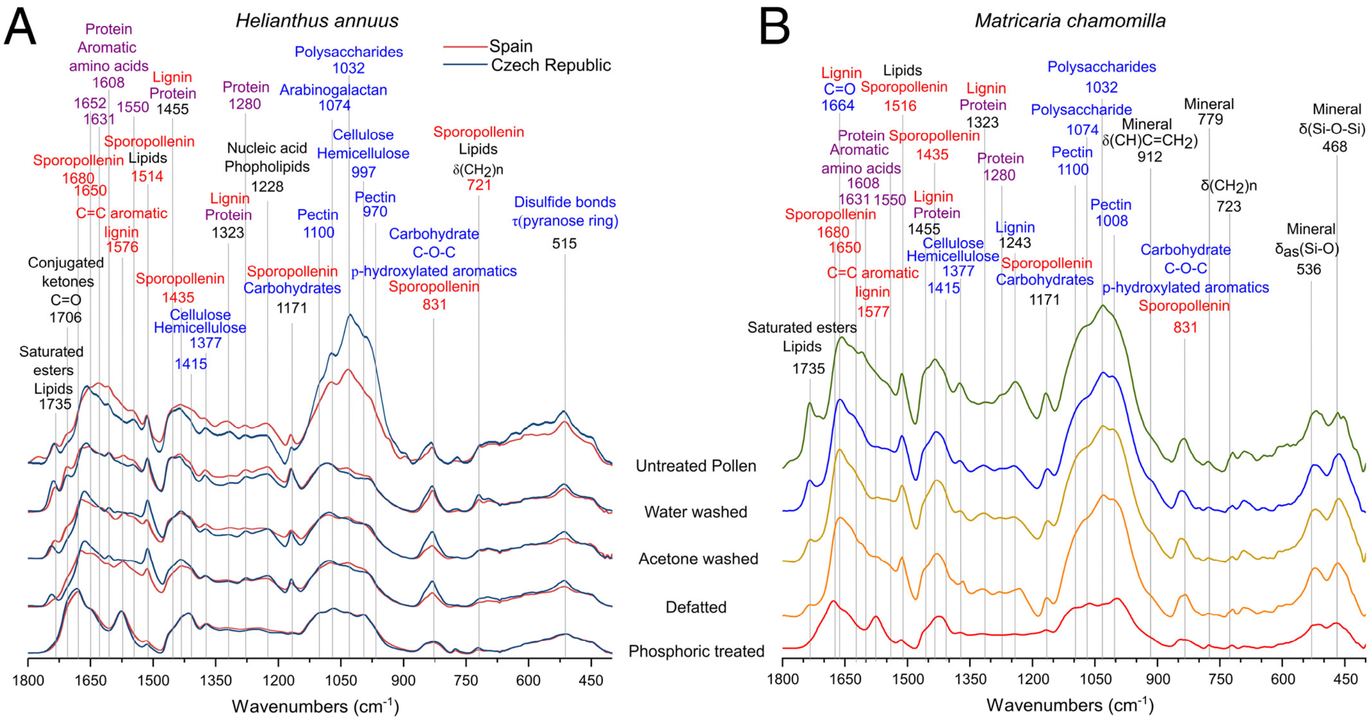

Figure S1. ATR-FTIR spectra of pollen samples. A. Comparison spectra of sunflower pollen from Spain and Czech Republic. B. Spectra of chamomile pollen treated with extended extraction protocol.

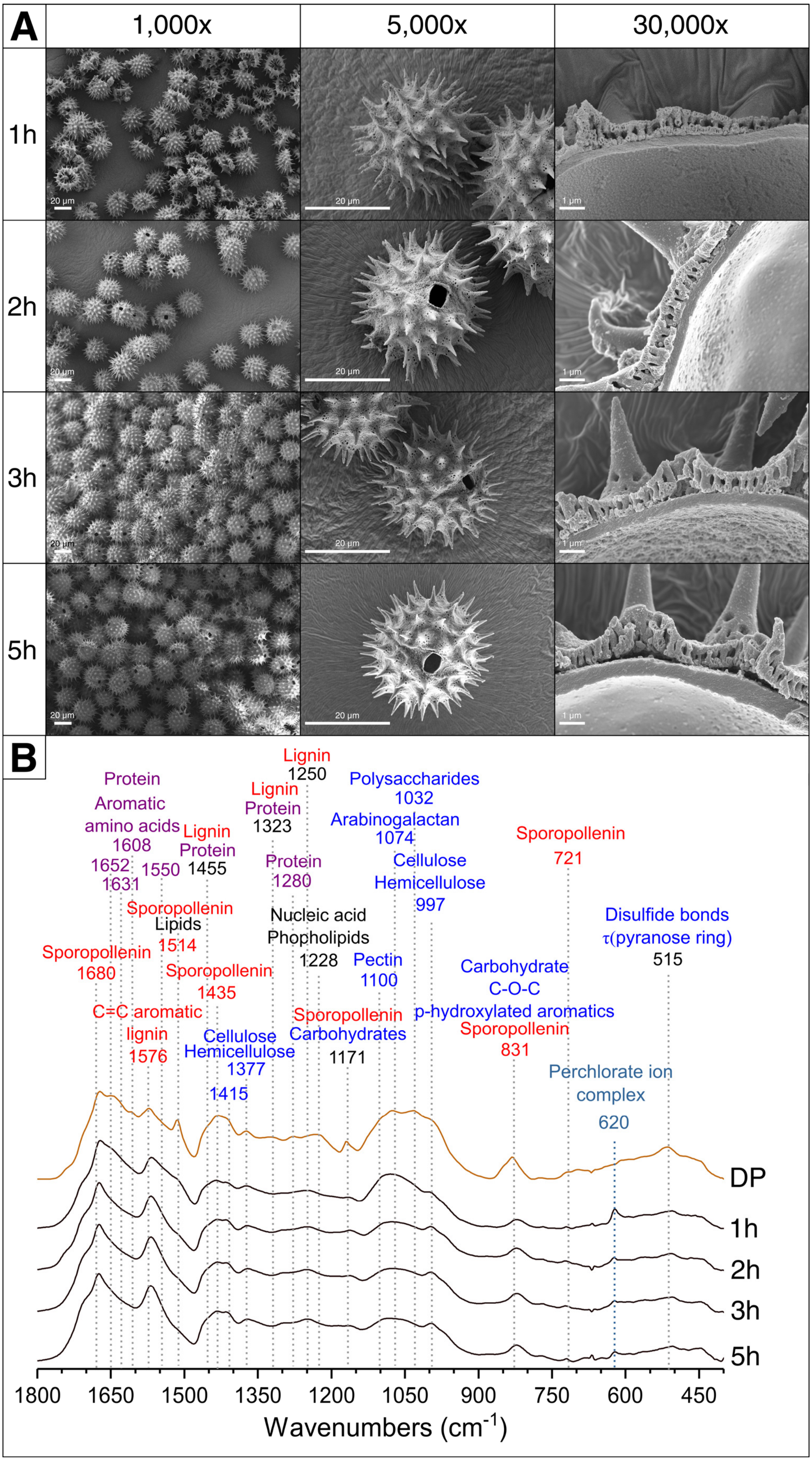

Figure S2. Time course study of the treatment of defatted sunflower (DP) treated with $HClO_4$ during different time periods. **A**. SEM images at different magnifications. **B**. ATR-FTIR spectra of pollen samples indicating the band assignment.

Table S1. Principal characteristics of sunflower and chamomile pollen samples.

	**Sunflower**		**Chamomile**	
	**Defatted**	**Hollow**	**Defatted**	**Hollow**
Total diameter (µm)	42.9 ± 1.9	35.0 ± 2.0	24.8 ± 1.1	20.2 ± 1.3
Inner diameter (µm)	-	24.7 ± 0.9	-	12.1 ± 0.7
Density (g/cm³)	0.38 ± 0.06	0.13 ± 0.01	0.44 ± 0.02	0.14 ± 0.01
Grains per gram	~9.0x10⁷	~2.5x10⁸	~5.1x10⁸	~1.0x10⁹
Grains per cm³	~3.4x10⁷	~3.3x10⁷	~2.2x10⁸	~1.4x10⁸
Nitrogen content (%)	6.42 ± 0.67	0.70 ± 0.13	3.52 ± 0.30	0.64 ± 0.21
Protein content (%)	40.1 ± 4.2	4.4 ± 0.8	22.0 ± 1.9	4.0 ± 1.3

Table S2. TGA results for sunflower and chamomile pollen samples.

**Sunflower pollen**								
**Untreated pollen**			**Defatted pollen**			**Hollow pollen**		
T_{onset} (ºC)	T_d (ºC)	Δw (%)	T_{onset} (ºC)	T_d (ºC)	Δw (%)	T_{onset} (ºC)	T_d (ºC)	Δw (%)
30-107	54	6	30-137	52	2	30-137	47	2
108-175	153	2	138-289	284	13	138-247	200	6
176-257	235	14	290-405	326	38	248-369	316	8
258-310	272	16	406-529	439	21	370-531	442	39
311-386	316	20	530-800	-	3	532-800	-	3
387-490	425	16	Ash		24	Ash		42
491-800	-	5						
Ash		20						
**Chamomile pollen**								
**Untreated pollen**			**Defatted pollen**			**Hollow pollen**		
T_{onset} (ºC)	T_d (ºC)	Δw (%)	T_{onset} (ºC)	T_d (ºC)	Δw (%)	T_{onset} (ºC)	T_d (ºC)	Δw (%)
30-99	56	2	30-111	52	3	30-111	47	2
100-175	145	5	112-285	270	11	112-352	304	16
176-244	218	8	285-382	325	24	353-550	451	41
245-282	270	6	383-507	448	21	551-800	-	2
283-361	308	15	508-703	674	6	Ash		39
362-429	404	8	704-800	-	3			
430-506	450	6	Ash		32			
507-590	515	3						
591-692	644	5						
693-800	-	2						
Ash		40						

T_{onset}; range of temperatures in the thermal decomposition. T_d: degradation temperature, Δw: variation of mass occurred in the T_{onset}.
