# Supplementary figures and images for: Purification of Hollow Sporopollenin Microcapsules from Sunflower and Chamomile Pollen Grains
Source: Polymers (Basel). 2021 Jun 25;13(13):2094. doi: 10.3390/polym13132094 (PMC8271440; doi:10.3390/polym13132094)

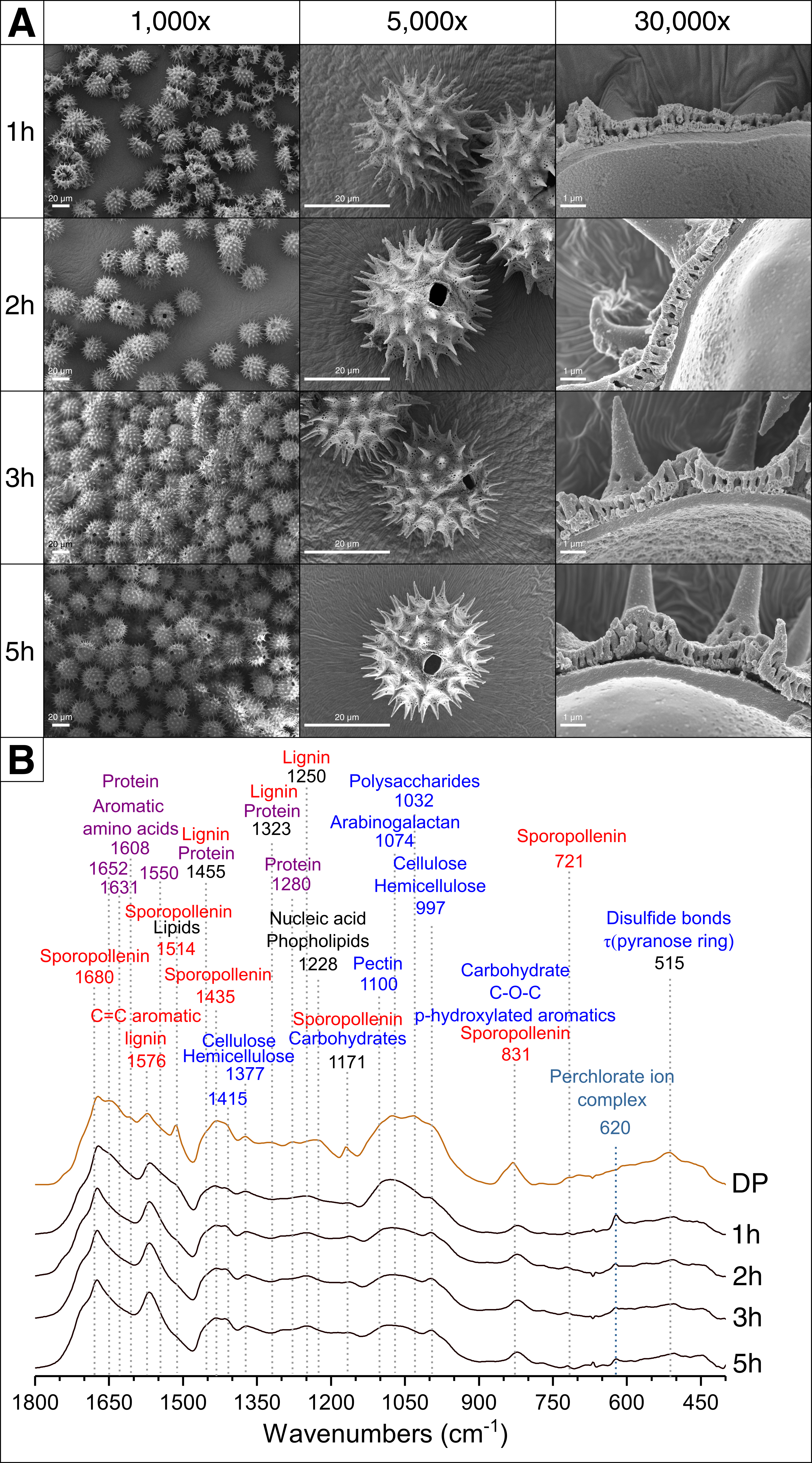

Supplement: Supplementary file 1 [file polymers-13-02094-s001.zip › FIGS2.JPG]
